# Physical Function Transitions and Healthcare Utilization Among Older Mexican Americans.

**DOI:** 10.1093/geroni/igab046.3186

**Published:** 2021-12-17

**Authors:** Amy Givan, Lin-Na Chou, Yong-Fang Kuo, Soham Al Snih

**Affiliations:** University of Texas Medical Branch, Galveston, Texas, United States

## Abstract

The aim of this study was to examine the relationship between 2-year physical function transitions and one-year healthcare utilization among Mexican American Medicare beneficiaries. The sample consisted of 429 Mexican Americans ≥75 years old from the Hispanic Established Population for the Epidemiologic Study of the Elderly linked with Medicare claims data from the Centers for Medicare and Medicaid Services. Short Physical Performance Battery (SPPB) from Wave 5 (2004/05) to Wave 6 was used to create physical function transition groups. The outcomes were physician visits (<6, 6-12, >12 visits), number of emergency room visits, and number of acute hospitalizations one-year after physical function transitions. Multinomial logistic regression and Generalized Estimating Equation with negative binomial distribution were used to estimate the odds ratio of healthcare utilization as a function of physical function transition groups, controlling for socio-demographics and comorbidities. Participants who improved or remained moderate-high physical function had lower odds ratio (OR) of being hospitalized (0.40, 95% Confidence Interval [CI]=(0.18, 0.90)) or visiting the emergency room (OR=0.52, 95% CI=0.32-0.84) one-year later compared to participants who remained in the low physical function group. No difference in physician visits across physical function transition groups was found. This study showed healthcare utilization differed by physical function transition groups among Mexican American Medicare beneficiaries. Physical function improvement or maintenance of moderate-high physical function should be targeted in older Mexican Americans, a population at great risk of developing disability, to reduce or delay dependency and healthcare burden.

